# Sertoli Cell Immune Regulation: A Double-Edged Sword

**DOI:** 10.3389/fimmu.2022.913502

**Published:** 2022-06-09

**Authors:** Rachel L. Washburn, Taylor Hibler, Gurvinder Kaur, Jannette M. Dufour

**Affiliations:** ^1^ Department of Cell Biology and Biochemistry, Texas Tech University Health Sciences Center, Lubbock, TX, United States; ^2^ Immunology and Infectious Disease Concentration, Texas Tech University Health Sciences Center, Lubbock, TX, United States; ^3^ Department of Medical Education, Texas Tech University Health Sciences Center, Lubbock, TX, United States

**Keywords:** Sertoli cells, immune regulation, transplantation, cell therapeutics, testis

## Abstract

The testis must create and maintain an immune privileged environment to protect maturing germ cells from autoimmune destruction. The establishment of this protective environment is due, at least in part, to Sertoli cells. Sertoli cells line the seminiferous tubules and form the blood-testis barrier (BTB), a barrier between advanced germ cells and the immune system. The BTB compartmentalizes the germ cells and facilitates the appropriate microenvironment necessary for spermatogenesis. Further, Sertoli cells modulate innate and adaptive immune processes through production of immunoregulatory compounds. Sertoli cells, when transplanted ectopically (outside the testis), can also protect transplanted tissue from the recipient’s immune system and reduce immune complications in autoimmune diseases primarily by immune regulation. These properties make Sertoli cells an attractive candidate for inflammatory disease treatments and cell-based therapies. Conversely, the same properties that protect the germ cells also allow the testis to act as a reservoir site for infections. Interestingly, Sertoli cells also have the ability to mount an antimicrobial response, if necessary, as in the case of infections. This review aims to explore how Sertoli cells act as a double-edged sword to both protect germ cells from an autoimmune response and activate innate and adaptive immune responses to fight off infections.

## Introduction

The primary function of the testis is to produce male gametes and testosterone. Spermatogenesis is first initiated when testosterone levels rise at puberty, long after immune self-tolerance has been established. Given that the majority of germ cells do not appear until this time, the testis must be able to prevent an immunological reaction to germ cells and autoimmune orchitis. At the same time, the testis must be able to mount a robust enough response to microbes to prevent infections, as inflammation can also lead to male infertility. If this does not happen, the testes can become a sanctuary for infections. Therefore, understanding the mechanisms for how the testis can both protect germ cells from an autoimmune response and fight off infections is important for male fertility. While several components of the testis contribute to this process, for this review we will focus on the role of the Sertoli cell.

### Testicular Immune Privilege

Primordial germ cells migrate into the indifferent gonad during embryonic development. In the male, Sertoli cells aggregate around these germ cells and mesenchymal cells, which journey from the mesonephros, surround the aggregates to form the seminiferous cords. The male germ cells undergo mitosis until late embryonic development, when they become quiescent, and resume proliferation after birth. Puberty triggers the onset of spermatogenesis, leading to the formation of the seminiferous tubules. Spermatogenesis includes three stages of germ cell development: mitosis (spermatogonial proliferation), meiosis (spermatocyte DNA recombination, reduction, and division) and spermiogenesis (spermatid differentiation). The appearance of these late-stage germ cells at puberty is immunologically significant as they express antigens that are not present during the development of immunological central tolerance, which occurs right after birth, and are therefore labeled as foreign (1). If detected by the immune system, these “non-self” antigens can trigger an autoimmune response—autoimmune orchitis—which is characterized by inflammation and anti-sperm antibodies. Autoimmune orchitis can lead to decreased fertility or complete infertility if left untreated. However, as the majority of males will not experience autoimmune orchitis the testes must have developed a way to prevent unwanted immunological attention.

Support for the immune-privileged status of the testis comes from transplantation studies where testis tissue fragments enjoy prolonged graft survival when transplanted between immunologically different individuals. This was first demonstrated by John Hunter in 1767 when he transplanted testes from roosters into hens (2); this discovery eventually led to the use of testicular extracts for their so-called rejuvenating effects in the early 1900s. Similarly, allogeneic or xenogeneic tissue survives for an extended period of time after transplantation into the testis (3). For example, transplantation of pancreatic islets from rats into testes in diabetic mice resulted in not only prolonged graft survival but also normalization of blood glucose levels (4).

However, the testes must also be able to provide an effective immune response to prevent microbial damage. Many viruses and bacteria can infect the testis and affect male fertility (5, 6). Approximately 8-15% of couples globally are infertile and 40-60% of them are due to male infertility caused by infection, trauma, environmental toxins, or varicoceles. Inflammation associated with these conditions can result in immune attack on the immunogenic germ cells; indeed, 5-12% of infertile males have sperm autoantibodies (7). Consequently, the testis needs to be able to mount an effective immune response to prevent infection or else it could become a sanctuary for viruses and bacteria.

The mechanism(s) responsible for immune protection in the testis have been an area of interest for some time [reviewed extensively in (7)]. Components such as lower scrotal temperature, elevated levels of zinc, impaired drainage to lymph nodes, spermatogenesis, immune cells (for example M2 macrophages and regulatory T cells), and testicular cells (germ cells, Sertoli cells, Leydig cells and peritubular myoid cells) have all been investigated for their potential involvement and, it was found that impaired lymphatic drainage, high zinc, and lower temperature are not critical components for testicular immune regulation [reviewed in (7)].

**Figure 1:**
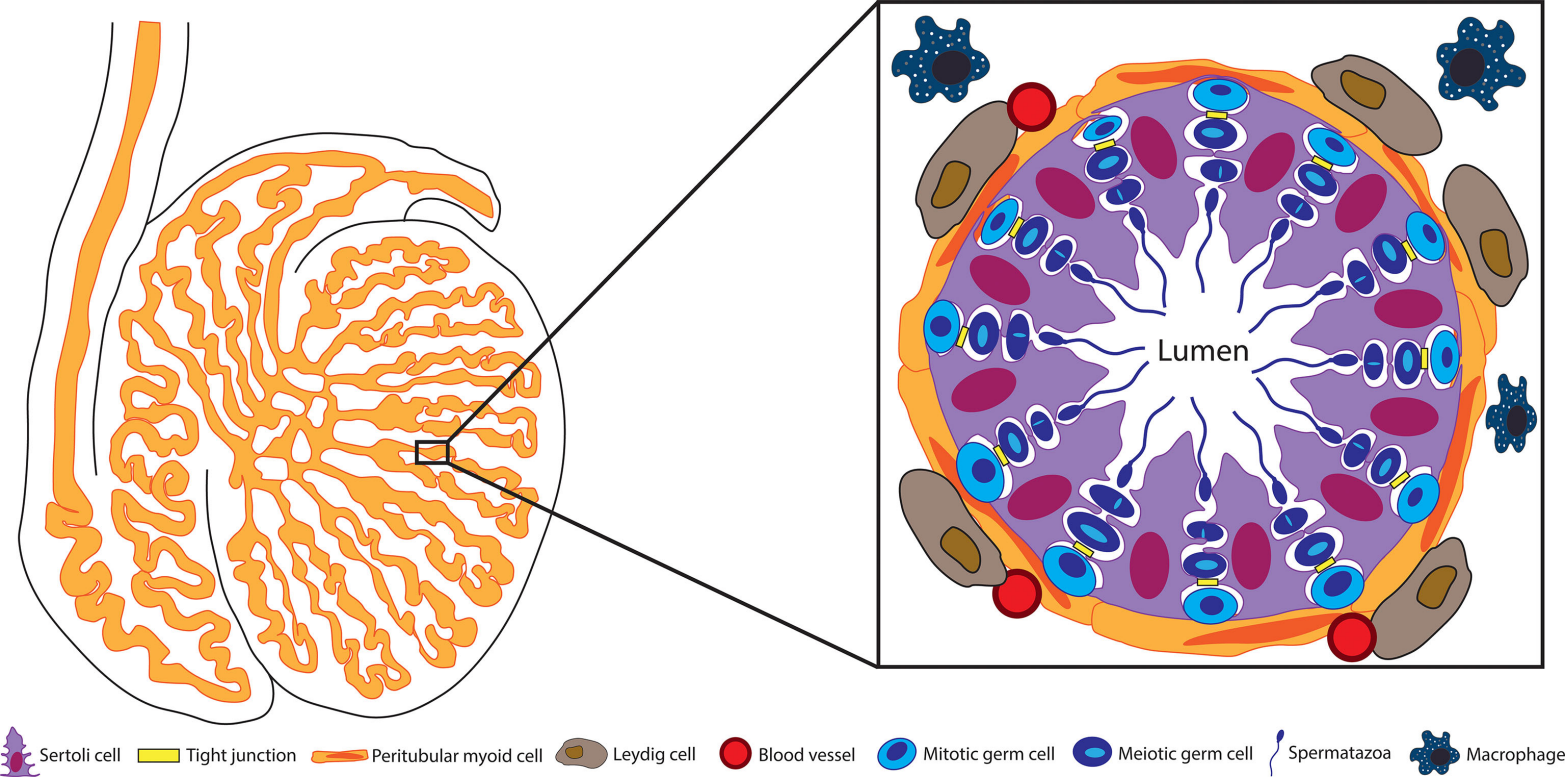
Structure of the seminiferous tubule. Spermatogenesis takes place within the seminiferous tubules. These tubules are surrounded by peritubular myoid cells and contain the germ cells engulfed by Sertoli cells. The BTB between adjacent Sertoli cells separates the seminiferous epithelium into the basal and adluminal compartments. Blood vessels, Leydig cells, and testicular macrophages are located outside the tubules in the testis interstitium.

The testis is comprised of seminiferous tubules separated by interstitium (**Figure 1**). The interstitial space contains Leydig cells, leukocytes, fibroblasts, blood and lymphatic vessels. The seminiferous epithelium is composed of Sertoli cells and germ cells surrounded by peritubular myoid cells. It seems that most of these cells contribute, at least in part, to the immunomodulatory environment of the testis.

Earlier studies demonstrated that Leydig cells are not essential for testicular immune regulation as both inhibition of testosterone production and depletion of Leydig cells did not affect the prolonged survival of intratesticular transplanted allogeneic pancreatic islets (8). On the other hand, testosterone supplementation provided protection from experimental autoimmune orchitis by inducing regulatory T cells (Tregs), decreasing inflammation, and decreasing macrophage and CD4 T cell infiltration (9). Moreover, Leydig cell conditioned media has been shown to inhibit lymphocyte proliferation (3) and more recently, induce Tregs (10). Thus, Leydig cells and their secreted products play a role in regulating the immune response.

Similarly, germ cells and spermatogenesis are not required for immune protection. Transplantation of islets or parathyroid grafts into the cryptorchid or irradiated testis still resulted in sustained graft survival despite the loss of germ cells and spermatogenesis (3). Then again, germ cell culture media inhibits lymphocyte proliferation and syngeneic germ cells injected into rodents reduce NK cell activity and inhibit a cytotoxic T lymphocyte response (11). Interestingly, germ cell transplantation between genetically different donor and recipient has been successful in mice, pigs, goats, cattle, dogs, cats, sheep, chickens, and fish without the addition of immune suppression, and resulted in live offspring in mice, goats, sheep, chickens, and fish (12–14); although it is not clear whether the immune protection observed was due to the germ cells and/or the somatic cell environment. Much like Leydig cells, germ cells and spermatogenesis may contribute to immune protection, but are not the sole purveyors of immune privilege.

There is some evidence that peritubular myoid cells may also contribute to the immune environment of the testis, although more work needs to be done with these cells to confirm their role. Peritubular myoid cells are located in close proximity to other testicular cells and, therefore, have the ability to respond and influence other testicular cells including immune cells (such as macrophages) (15). They produce several immunomodulatory factors (e. g., transforming growth factor (TGF)-β, B7-H1, interleukin (IL)-6 and monocyte chemoattractant protein (MCP)-1) and have been found to respond to immune cell products like tumor necrosis factor (TNF)-α (15). Cell ablation studies support the importance of the peritubular myoid layer in preventing entry of immune cells into the seminiferous tubules, as after ablation of Sertoli cells and loss of germ cells, the remaining peritubular myoid cells were sufficient to inhibit macrophage entry into the tubules (16, 17).

Several studies have demonstrated that Sertoli cells are important for testicular immune regulation (5, 7). Ablation of Sertoli cells confirmed the role of these cells in testis and germ cell development and formation of the blood-testis-barrier (BTB), which prevents entry of molecules, antibodies and immune cells into the tubules. It was also found that Sertoli cells are important for adult Leydig cell survival and peritubular myoid cell function (16, 17). Additionally, Sertoli cells survive long-term after transplantation as allografts or xenografts to ectopic sites without the use of immunosuppressive drugs (18). Moreover, Sertoli cells prolong the survival of co-transplanted allogeneic and xenogeneic tissues, such as pancreatic islets, suggesting that Sertoli cells can mimic the immune privileged environment outside of the testis (18). Sertoli cells also produce several immune modulatory factors (discussed later) that influence the immune response. Finally, after depletion of Leydig cells and germ cells (as described above), tissues transplanted into the testis were not rejected. As Sertoli cells are the main testicular cell remaining, this implicates them in this protection.

It seems clear that testis immune privilege is due to the contributions of several cell types within the testis. This review will focus on the role of Sertoli cells in modulating the innate and adaptive immune responses to simultaneously provide germ cell immune protection and yet also inhibit infections.

### Immune Regulation in the Testis

**Figure 2:**
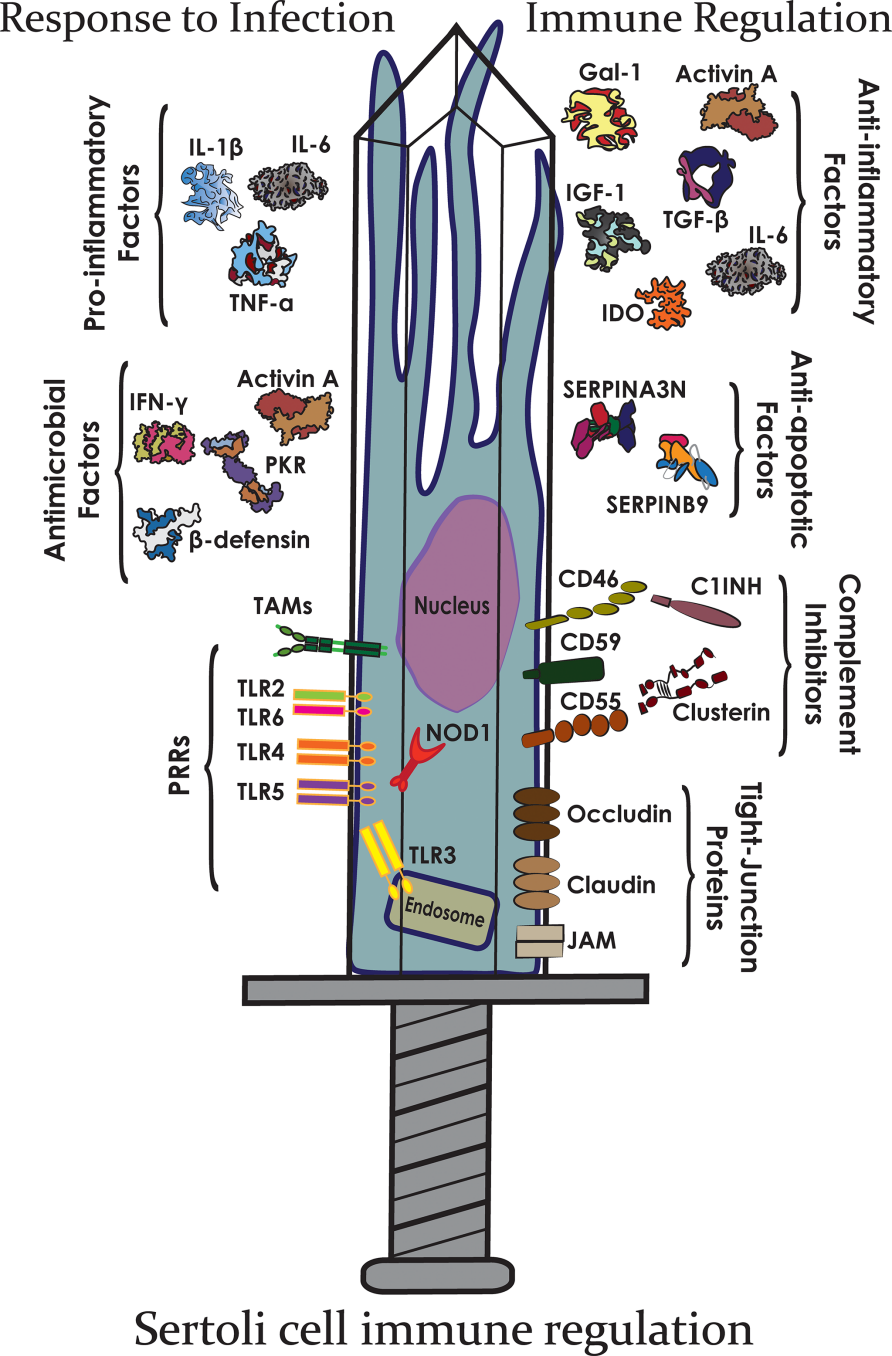
The Sertoli cell double-edged sword. Sertoli cells express many different immunomodulatory factors that allow them to protect germ cells from both infection and immune responses.

The testis must maintain a delicate balance between preventing an unnecessary immune response against auto-immunogenic germ cells and encouraging a preventative response against pathogens and tumor formation. To achieve this balance, the testes must regulate both arms of the immune response: the immediate, non-specific response of innate immunity (19) and the controlled, antigen-specific response of adaptive immunity (20), briefly described below (**Figure 2**).

**Figure 3:**
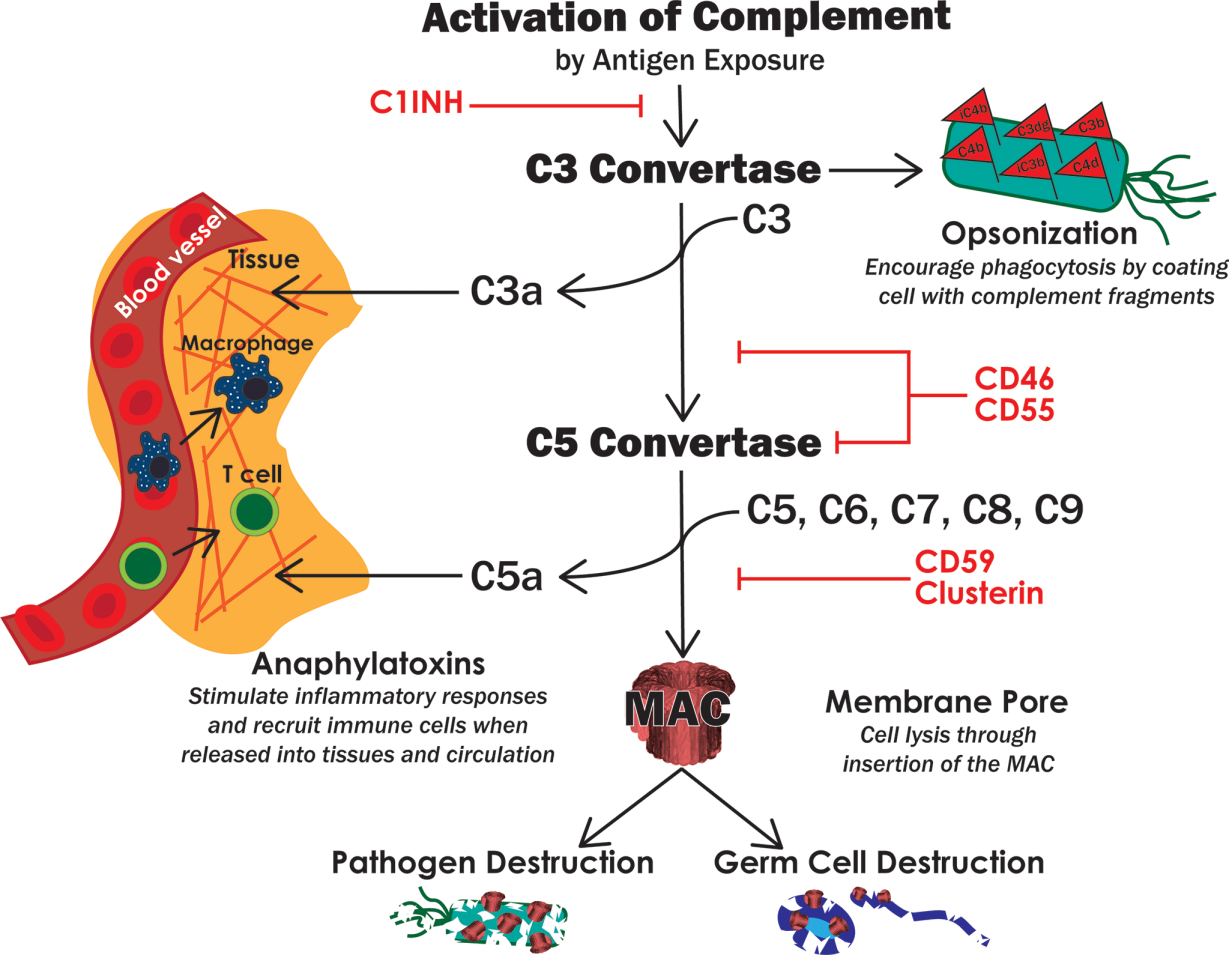
The complement system. Activation of complement leads to the formation of two convertases, which then lead to opsonization of target cells, secretion of proinflammatory anaphylatoxins and insertion of the MAC pore. These functions of complement work together to destroy pathogens but can also destroy host cells if not properly regulated. Red text indicates known complement inhibitors expressed by Sertoli cells.

The innate immune system is the first line of defense and involves early, rapid, and non-specific responses to pathogens (19). These include biochemical, protein, and cellular components that are already in place and ready to respond when a threat is detected. Innate immunity includes components such as: physical barriers, antimicrobial proteins, the complement system, and innate immune cells. Physical barriers, like the epidermis and epithelial cells that line the alimentary canal, tend to be epithelial layers that prevent microbes from entering the body. In addition to a purely physical obstacle, these cells can secrete antimicrobial compounds and other proteins in a mucosal membrane to further prevent microbial invasion. If microbes penetrate these barriers, the next layer of protection to be activated is the complement system. Complement is a series of enzymatic proteins that culminate with lysis of the foreign tissue by insertion of an intermembrane pore (membrane attack complex, MAC) in addition to an intense inflammatory response through anaphylatoxin release and recruitment of leukocytes (**Figure 3**) (21). Phagocytic leukocytes, such as monocytes, macrophages, and dendritic cells, are then rallied in defense of the host cells. These cells engulf and destroy foreign tissue, release cytotoxic molecules, and present antigens to adaptive immune cells (22). They can also produce antimicrobial proteins like ILs, TNFs, defensins, and activins, that combat pathogens (23).

The adaptive immune system involves a slower, highly specific, and expanded response to foreign tissue (20). Adaptive immunity includes the humoral response (B cells) and the cellular response (T cells). B and T cells must be activated to initiate their responses. Antigens are presented by antigen presenting cells (APC), phagocytic cells that display parts of the pathogen on their major histocompatibility complexes (MHC) for lymphocyte recognition (22). T cells, for instance, can be activated by macrophages, dendritic cells, and B cells. Once activated, T cells perform various functions depending on their subtype; that is if they are CD4+ helper T cells (Th) or CD8+ cytotoxic T cells (CTLs). Th cells activate and sustain both adaptive and innate immune responses, including activating macrophages to phagocytose microbes, instructing CTLs to destroy foreign or viral infected cells, and influencing B cells to differentiate into antibody-producing plasma cells. T cells and B cells, like macrophages and dendritic cells, also release antimicrobial and inflammatory peptides.

There is much overlap between the innate and adaptive immune systems (24). Cells from both systems can secrete inflammatory peptides such as interferons (IFNs), TNFs, and ILs, which can then go on to activate other immune cells (20). This generates a positive feedback loop that expands the total number of cells involved in the immune response to infection. Furthermore, in addition to recruiting phagocytes, T cells, and B cells, the innate complement system can also be activated by antibodies produced by the adaptive immune plasma cells. Complement factors play an important role in the differentiation of proinflammatory Th1 cells and in the activation of B cell phagocytosis (21). Conversely, during contraction of the immune response, regulatory T cells (Tregs) can suppress the function of innate and adaptive effector cells by releasing suppressive cytokines or inducing effector T cell death directly (25, 26). Additionally, complement inhibitors and receptors can generate immune suppressive Tregs during contraction of immune responses (27). T cells and B cells both express many complement inhibitory proteins that can effectively shut down the inflammatory responses generated by complement during contraction of the immune response (27). Taken together, this interplay between innate and adaptive immunity is crucial to coordinate, expand, and contract inflammatory immune responses to foreign tissue, and in protecting host cells from collateral damage in the process.

## Sertoli Cell Protective Response in the Testis and Summary of Potential Therapeutic Applications

As detailed above, Sertoli cells are a major player in testis immune regulation. The following section will examine the interactions between Sertoli cells and various components of the immune system. Given the complexities of the immune system, we will not discuss everything but will provide specific examples in the areas most relevant to Sertoli cells.

### Blood-Testis-Barrier

Generally, blood tissue barriers control the entry of macromolecules and cells from the blood into tissues. For most tissues this barrier is localized to the tight junctions of endothelial cells lining the blood vessels; however for the testis, while there is some evidence to suggest that there is a delay in the entry of substances into the interstitium from the vasculature (3), the primary BTB is located within the seminiferous tubules (**Figure 1**). The BTB proper consists of the body of the Sertoli cells and the tight junctions (TJ) formed between the Sertoli cells, although the peritubular myoid cells also contribute. In rodents, but not higher mammals, the peritubular myoid cells provide a semi-permeable barrier by decreasing the entry of large molecules in 85-90% of tubules (28) and preventing entry of leukocytes (16), while the Sertoli cells create a barrier impermeable to macromolecules and immune cell entry.

The primary function of the BTB is to control the passage of molecules and cells into the lumen of the seminiferous tubules. This allows the barrier to the create the appropriate microenvironment for germ cell development and maturation, protect the germ cells from exposure to toxins and prevent activation of an autoimmune response to the novel germ cells antigens. There are three components to the BTB, the physical, physiological, and immunological, that work together to achieve this goal.

The physical and physiological components cooperate to control the movement of substances across the barrier. In this way the Sertoli cells create a microenvironment conducive to development (meiosis and differentiation) and maturation (spermiogenesis) of germ cells. The physical barrier restricts the passage of molecules across the BTB. The functional BTB includes the TJ, basal ectoplasmic specializations, gap junctions and desmosomes. Unique to the testis, the TJ are located toward the basal edge of the Sertoli cells and divide the seminiferous epithelium into the basal and adluminal compartments. The physiological part of the BTB includes specific transporters located along the basal and apical membranes of the Sertoli cells that regulate the movement of substances across the barrier; from the interstitium to the lumen and also from the lumen to the interstitium (28).

The immunological part of the BTB acts together with the peritubular myoid cells to prevent entry of leukocytes into the seminiferous tubules. It also sequesters the autoantigenic germ cells from contact with the immune system and blocks antibodies and other immune factors from freely entering the adluminal compartment. Measurement of fluid collected from the rete testis lumen found that immunoglobulin levels were about 0.2% of those found in serum and similar measurements of seminiferous tubule fluid found undetectable levels of immunoglobulins (29, 30).

While it is correct that the BTB sequesters the majority of the autoantigenic germ cells, sequestration of germ cell antigens is a bit more complicated. Recently, Tung et al, reported that germ cell antigens can be categorized as either sequestered or non-sequestered (1). The sequestered germ cell antigens behave as expected as they are not tolerogenic and are protected from an immune response only by the BTB. On the other hand, the non-sequestered germ cell antigens are exported as cargo in residual bodies where they are present in immune complexes within the interstitium. These exported antigens induce Treg dependent tolerance and depletion of Tregs leads to a serum antibody response and autoimmune orchitis directed against the non-sequestered antigens.

Thus, the BTB is an important part of testis immune privilege. However, contrary to popular belief, it is not the sole element responsible for this immune privilege (7). For example, allogeneic or xenogeneic tissue transplanted into the testis enjoys prolonged grafts survival even though it is located outside of the BTB in the interstitial space. Additionally, preleptotene spermatocytes express auto-immunogenic antigens and are located outside of the BTB, and the BTB repeatedly breaks down and reforms in seasonal breeders exposing meiotic germ cells (1, 31) and yet neither situation evokes an immune response. Instead, other mechanisms of immune privilege are involved. These mechanisms, especially how they relate to the Sertoli cells will discussed further in this review.

### Complement System

One mechanism that Sertoli cells must modulate to prevent spermatocyte destruction is the complement system. The complement system is one of the first innate immune components activated upon pathogen exposure (32). Complement is a series of plasma immune proteins that, after activation, eventually leads to pathogen destruction by insertion of an intermembrane pore (MAC), inflammatory responses, and opsonization for phagocytosis (**Figure 3**). Complement is primarily activated through three pathways: classical (antibody binding), lectin (bacterial oligosaccharides), and alternative (spontaneously or C3 cleavage) (32).

All three activation pathways converge at the development of a C3 convertase, whose function is to bind and cleave complement component C3 (32). The anaphylatoxin C3a is released, which allows for modulation of inflammation and recruitment of leukocytes *via* chemotaxis (33). C3b binds to the pathogen, marking it for phagocytosis by immune cells, or to bind to the C3 convertase thus forming the C5 convertase. As more C3 is cleaved, C3b and factor B can continue to form C3 convertases to amplify the complement response (33). Thus, C3 convertase formation is the pinnacle of complement amplification as it is the basis of a positive feedback loop. The C5 convertase binds complement component C5 to release the anaphylatoxin C5a. C5b now can bind C6, C7, C8, and C9-C8 forming the MAC and lysing the target cell (32, 34).

As complement has the potential to be amplified and operates by a positive feedback mechanism, there is great potential for collateral damage to host cells. Moreover, if immunogenic germ cells are exposed to humoral components such as antibodies, complement can become activated within the seminiferous tubules. This leads to germ cell destruction, infertility, and/or even autoimmune orchitis. To prevent unwanted destruction and inflammation, host cells express complement inhibitors (34). To date, Sertoli cells have been shown to express the following complement inhibitors: C1 inhibitor (C1INH, SERPING1), CD46 (membrane cofactor protein, MCP), CD55 (decay accelerating factor, DAF), CD59, and clusterin (**Figure 3**) (35–37). C1INH is a soluble protein that binds to and dissociates the activation phase C1 complex, thus decreasing further activation along the complement cascade (37, 38). CD46 and CD55 each inhibit both C3 convertase and C5 convertase which decreases complement amplification, MAC formation, and anaphylatoxin levels (39). CD59 and clusterin inhibit assembly of the MAC and thus directly inhibit cytolysis (40, 41). Altogether, these complement inhibitors act throughout the entire cascade to effectively shut down complement-mediated cell lysis, opsonization, and inflammation. In fact, we have previously shown that Sertoli cells inhibit complement before MAC insertion using a human serum and complement cytotoxicity assay (42). Similar results were observed *in vivo* where neonatal pig Sertoli cells after transplantation under the kidney capsule of nonimmune suppressed rats inhibited complement activation, specifically C3 deposition and MAC formation (36). At the same time there was reduced migration of APCs and increased Treg migration that may be at least partly due to the presence of fewer anaphylatoxins (C3a and C5a) (43). CD59, CD46, CD55, and CD59 also play important roles in sperm development, transit through male and female reproductive tracts, and fertility (44). CD46 is a component of the acrosomal membrane on sperm, which stabilizes the acrosome and prevents premature acrosomal degradation, which may be important for successful sperm-egg fusion and aid in clearance of residue sperm in seminiferous tubules (45). In human males with decreased CD46 expression, fertility is hindered (46). Sperm are also coated on the tail, neck, and acrosome by the complement inhibitors CD55 and CD59, which protects sperm from female complement-mediated destruction and may play other roles in fertilization (45, 47). This is further supported as loss of the *CD59b* gene in mice led to a loss in male fertility (48). Overall, complement proteins are important in male fertility and mounting an effective immune response against pathogens, modulating inflammation, and recruiting and activating innate and adaptive immune cells.

### Immune Environment

Macrophages, dendritic cells, T cells, and B cells are all immune cells that can affect testicular function. Of these, macrophages are the primary immune cell found within the testis. Sertoli cells express several immunomodulatory factors (**Table 1**) and can influence the action and function of these different immune cells to maintain protection of the immunogenic germ cells. This will be discussed now in more depth.

**Table 1:** Selected immunomodulatory factors expressed by Sertoli cells.

Cytokine	Protects from	Immune function	Function in testes	Ref
**Gal-1**	Immune system	Encourages development and maintenance of tolerogenic dendritic cells	May play a role in spermatogenesis and spermiation	(49, 50)
**IGF-1**	Immune system	Decreases production of proinflammatory cytokines IL-1β, TNF-α, and leukocyte chemoattractants	Cell development, proliferation, and function	(51–53)
**IDO**	Immune system	Suppresses effector T cell functions, induces tolerogenic APCs and Tregs	Testicular immune tolerance, protects against autoimmune orchitis	(54, 55)
**TGF-β**	Immune system	Develops immune tolerance to self and generates Tregs	Regulates Sertoli cell TJ, encourages germ cell differentiation and proliferation	(56, 57)
**Activin A**	Immune system	Aids in Th2 and Treg induction	Regulates FSH production and Sertoli cell and germ cell growth	(58–61)
**SERPINA3N**	Immune system	Inhibits granzyme B and CTL-mediated killing	Prevents apoptosis, may facilitate migration of developing germ cells	(62–64)
**SERPINB9**	Immune system	Protects cells from lymphocyte-secreted granzyme B	Anti-apoptotic cell survival factor	(65–70)
**IL-6**	Immune system and Infection	Increases production of acute phase proteins, platelet, and antibody production; and generates effector T cells; generates Tregs in the presence of TGF-β, retinoic acid, and dendritic cells	Mediates spermatogenesis signaling cascades	(71, 72)
**IL-1β**	Infection	Increases expression of proinflammatory cytokines TNF-α and IL-6, generates Th17 cells	Spermatogenesis maintenance	(73)
**TNF-α**	Infection	Inflammatory cytokine that leads to apoptosis or necrosis of cells	Promotes cell survival; regulates BTB with TGF- β in spermatogenesis to allow migration of spermatocytes	(74–76)
**β-defensin**	Infection	Antimicrobial peptide	Protects seminiferous tubules from infection	(77, 78)
**IFN-γ**	Infection	Activates effector immune cells like macrophages to clear viral infections; increase PKR expression, polarize T cells to Th1 cells	Regulates Sertoli cell survival and function, protects seminiferous tubules from viral infection	(79, 80)
**PKR**	Infection	Inhibits translation of viral mRNA, stimulates apoptosis of virus-infected cells, increases expression of IFN-γ	Protects seminiferous tubules from viral infection	(81, 82)

#### Macrophages

Many of the above-mentioned complement inhibitors, cytokines, and receptors expressed by Sertoli cells play a part in modulation of various leukocytes. Macrophages are phagocytic cells that primarily function to ingest pathogens and cellular debris. They are also part of both the innate and adaptive immune responses. As innate immune cells, macrophages will phagocytose anything they recognize as non-host cells or as damaged host cells. Macrophages also release a myriad of cytokines involved in immune cell activation, differentiation, proliferation, survival, chemotaxis, and recruitment (83). Macrophages are big players in clearance of dead cells, bacteria, infected cells, and in wound healing. After ingesting cells or cellular debris, macrophages present peptides from the cell fragments to T cells, adaptive immune cells, for further response (22, 83). By doing so, macrophages can activate T cells to proliferate and expand, creating an effective army of lymphocytes that can mount a strong and specific immune response against the invading pathogen (83).

Immune cells in healthy testes are only found in the interstitial space, blood vessels, or lymphatics, and not in the seminiferous tubules (22). Macrophages have been found to be the dominant immune cell found in the testis, making up about 20% of cells found in the interstitial space (84). At homeostasis, M2 macrophages, an immunoregulatory subtype, predominate over M1 macrophages, a more inflammatory subtype (22). Whereas M1 macrophages secrete inflammatory cytokines such as IL-1, TNF-α, and nitric oxide (22), M2 macrophages produce these at significantly lower levels while expressing very high levels of the immune suppressant cytokine IL-10 (85). It is known that macrophages can shift between M1 and M2 subtypes based on the local microenvironment. For instance, when macrophages are exposed to a more inflammatory environment, such as an infection or wound, M2 macrophages can convert to M1 macrophages to mount an effective immune response. However, if this inflammatory environment is sustained in the testes, it can lead to breakdown of the BTB and impaired spermatogenesis, thus maintaining the delicate balance of suppressive versus effector responses is critical in male reproductive health (22, 83). In addition to their immune duties, testicular macrophages aid in testosterone production by transferring cholesterol directly to the androgen-producing Leydig cells located within the testicular interstitium (86). Macrophage ablation studies further show that macrophages are essential for Leydig cell development (87); however, after Leydig cells are established, they are no longer critical for survival but enrich steroidogenesis (88).

**Figure 4:**
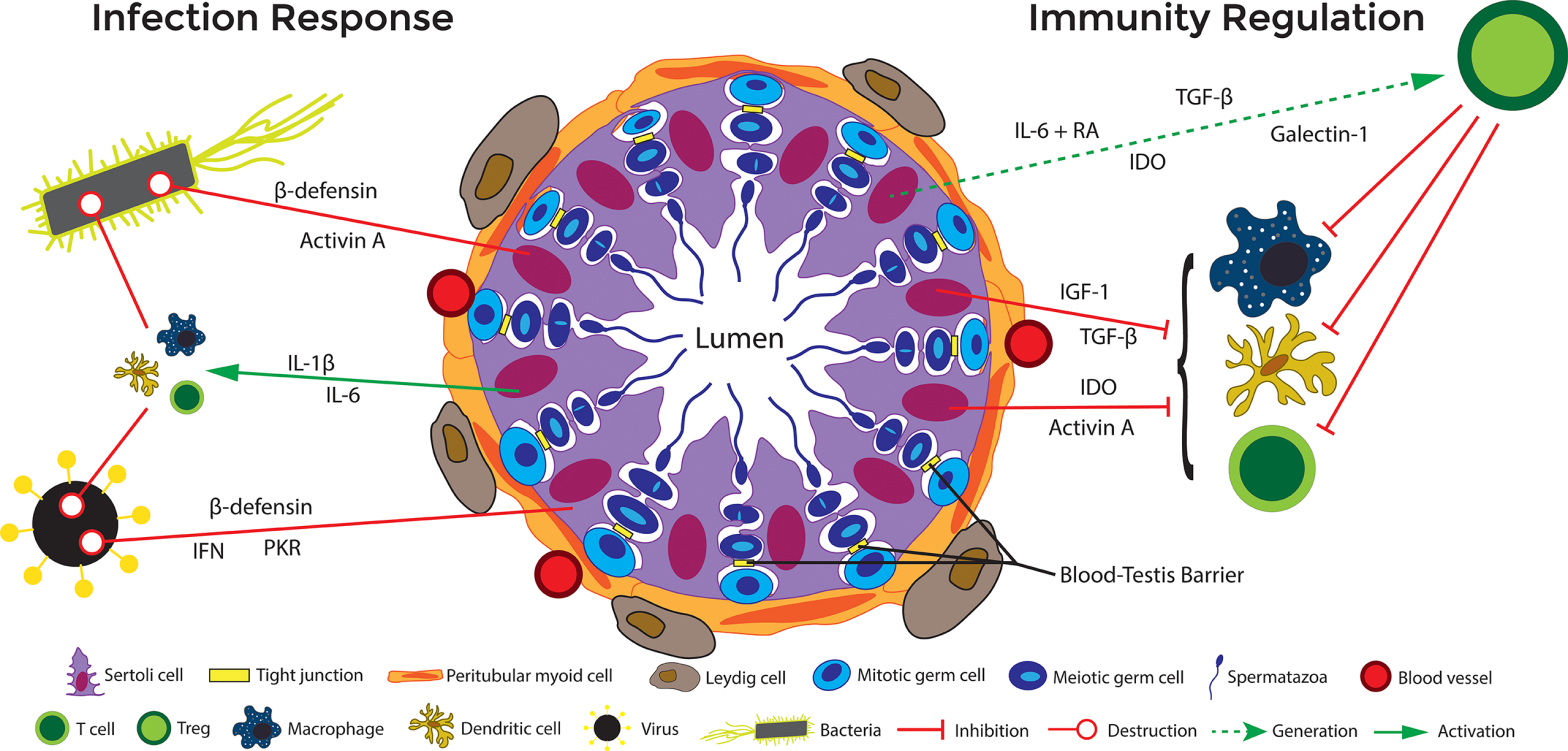
Immune regulation in the testis. Under normal circumstances, cells in the testis (including Sertoli cells) produce immunoregulatory factors that favor the presence of regulatory immune cells, including M2 macrophages, Tregs, and tolerogenic dendritic cells, that maintain a milieu supporting germ cell survival. On the other hand, if bacteria and viruses invade the testis, to eliminate the pathogen, the testis can initiate an inflammatory response that includes activation of PRRs, production of inflammatory cytokines and antimicrobial factors, and recruitment and activation of inflammatory immune cells such as M1 macrophages and cytotoxic T cells. If this response to infection is kept in check it does not disrupt normal testicular function and the testis is able to maintains a delicate balance to promote spermatogenesis and prevent infection. However, if it gets out of control, inflammation in the testis can lead to disruption of the BTB and loss of germ cells. At the same time, if the response is not strong enough to eliminate the infection, it can lead to a sanctuary reservoir for viruses and bacteria. Solid red lines indicate inhibition and dashed green lines indicate potential (but not proven) functions.

Sertoli cell secretion of activin A and TGF-β act to maintain an anti-inflammatory immunoregulatory environment conducive to preserving the M2 phenotype (**Figure 4**) (22, 83). In particular, activin A, a ligand in the TGF-β family, is critical for testis development (89), and also seems to influence the ratio of M1/M2 macrophages. When active A is increased in the testes, the ratio of CD206+ M2 macrophages to non-M2 macrophages is correspondingly increased (90). Furthermore, this immunosuppressive macrophage releases constitutive levels of IL-10, which encourages and generates immunosuppressive Tregs that continue in maintaining the immune privileged environment of the testes (84).

#### Dendritic Cells

Dendritic cells are APCs that play an important role in bridging the gap between the innate and adaptive immune system (91). The presence of tolerogenic dendritic cells have been noted in the rat testes before (92), but a recent study in mouse found that Sertoli cells secreted galectin-1 is important for development and maintenance of tolerogenic dendritic cells (49). When co-cultured with allogenic Sertoli cells, dendritic cells remain in an immature state, expressing lower levels of surface markers major histocompatibility complex II (MHC II, I-A/E), C-C motif chemokine receptor (CCR) 7, cluster of differentiation (CD)11c, CD80, CD83, and CD86. At the same time, they show increased immunoregulatory functions and secretion of large amounts of modulatory cytokines such as TGF-β and IL-10. Indeed, they have been shown to decrease T cell proliferation and increase CD4+ CD25+ Foxp3+ Treg cell differentiation.

The same has been shown in a xenogeneic co-culture of dendritic cells from C57BL/6 mice with neonatal pig Sertoli cells after exposure to lipopolysaccharide (LPS), a potent inflammatory antigen (93). Normally, after exposure to LPS, dendritic cells would upregulate the expression of MHC II (I-A/E) and T cell licensing molecule CD40. However, in the presence of neonatal pig Sertoli cells, the dendritic cells maintained basal levels of MHC II and CD40 expression and did not switch to an inflammatory phenotype. The ability of Sertoli cells to shift the dendritic cell response to a less inflammatory, more regulatory phenotype could have far reaching consequences in the testicular immune environment as tolerogenic dendritic cells encourage an anti-inflammatory T cell population.

#### T Cells

T cells make up about 10-20% of the immune cell population of the testes in humans and rats, and an increase in the number of T cells is associated with inflammatory pathologies (94). All major subsets of T cells have been found in human, rat, and mouse testes, as reviewed in (95). However, researchers have just begun to identify T cell interactions and functions in the non-inflamed testes, and it appears Sertoli cells may be a key component in influencing their behavior.

Several studies note that Sertoli cells appear to regulate the number of CD4+ and CD8+ T cells and activate Tregs to maintain immune homeostasis in the testes (91). In Sertoli cell/islet allografts, BALB/c islets and Sertoli cells were transplanted into both B6 and C3H mice and surviving grafts had a lower presence of macrophages, and CD4 and CD8 T cells overall. However, rejecting Sertoli cell/islet co-grafts showed increased numbers of macrophages and T cells (96). These results are mirrored in xenotransplantation experiments, in which ectopically grafted neonatal pig Sertoli cells were able to reduce the overall number of macrophages and CD3 T cells, and increase the number of Tregs (43).

The exact mechanism for how Sertoli cells limit T cell expansion remains somewhat of a mystery, however, it is known that one way that Sertoli cells limit the action of CD8+ CTLs is by expressing serine protease inhibitor (SERPIN)-A3N (novel granzyme B inhibitor) and SERPIN-B9 (protease inhibitor 9, cytoplasmic inhibitor of granzyme A and B) (62, 63, 65). CD8+ CTLs are the main effectors of T cell mediated damage in any tissues, and granzyme B is one of the main mechanisms through which CTLs destroy target cells. In addition to this immune modulatory function, SERPINA3N also inactivates trypsin, chymotrypsin, and cathepsin G to increase wound and tissue healing (63, 64). Another study found that Sertoli cells exposed to IFN-γ prevent CD8+ CTL destruction of target cells in co-culture by upregulating B7-H1 on their surface (97). Moreover, it has been shown that Sertoli cells grown in the presence of follicle-stimulating hormone (FSH) are able to inhibit the production of IL-2, a cytokine necessary for T cell proliferation (98).

In addition to reducing the number of inflammatory effector T cells, Sertoli cells can produce factors that skew all effector T cells to develop a more regulatory phenotype. Analysis of Sertoli cell gene expression by microarray revealed that they express TNF Receptor Superfamily Member 21 (TNFRSF21) and IL-1 Receptor Associated Kinase 3 (IRAK3), which push naïve T cells along the path of Th2 cells and Tregs, respectively (35). Thus, in both allo- and xenotransplantation models, Sertoli cells have been shown to reduce the number of T cells overall.

Another way that Sertoli cells alter the T cell population is through induction of Tregs. Tregs are a regulatory subset of T cells associated with repression of inflammation and tissue survival. *In vitro*, it has been shown that allogeneic mouse Sertoli cells induce Tregs from naïve T cells after exposure to inflammatory cytokine IFN-γ (97). Another allogenic *in vitro* study reported that soluble protein JAGGED1, secreted by mouse Sertoli cells, is critical in the induction of Tregs from naïve cells (99). Data from an ectopic xenogeneic transplant model shows that neonatal pig Sertoli cells not only survived transplantation into rats but increased the number of CD4+ and CD8+ Tregs (43, 100). The exact mechanism by which Sertoli cells induce Tregs has yet to be fully elucidated, but research indicates that Sertoli cells either directly [immunomodulatory factors such as TFG- β and indoleamine 2,3-dioxygenase (IDO)] or indirectly through other APCs (expression of galectin-1, activin A, TGF- β) induce Tregs (**Figure 4**). Additionally, microarray showed increased thrombospondin expression by Sertoli cells as compared to rejecting control cells (mouse Sertoli cell line), which can cleave inactive TGF-β into active TGF-β (35). TGF-β is required for the most common signaling pathways leading to Tregs. Additionally, the conditioned media from mouse Sertoli cells has been shown to induce functional CD4 Tregs *in vitro via* a TGF-β dependent manner. TGF-β and IDO have been implicated in Sertoli cell protection of transplanted syngeneic islets and reversal of diabetes in autoimmune non-obese diabetic (model of type 1 diabetes) mice, respectively (101). SERPIN-B9 has also been suggested to be key in survival of certain immune cells during both inflammatory and anti-inflammatory responses, such as dendritic cells (68, 69), Tregs (70, 102), and myeloid derived suppressor cells (102, 103) suggesting in addition to escaping C8 CTLs attack, Sertoli cells could be utilizing this molecule to induce regulatory cells. Overall, there is substantial evidence that Sertoli cells influence effector T cells and encourage the sustained function of Tregs.

#### B Cells

B cells phagocytose cells and present antigens to T cells to elicit immune responses against the antigen source. When activated by B cell receptor antigen crosslinking and helper T cells, B cells differentiate into antibody-producing plasma cells that secrete highly specific antibodies to bind to and mark foreign tissue for immune destruction. Outside the scope of antibody and cytokine production, little is known about Sertoli cell-B cell interactions. What is known is that B cells are not usually present in the healthy testes (104, 105). Also, proteins secreted by Sertoli cells grown *in vitro* have been reported to inhibit B cell proliferation (91, 106). Furthermore, B cells cocultured with xenotransplanted Sertoli cells demonstrated an increase in IL-10 production (107). As B cell research evolves to focus more on the lymphocyte itself, more information on Sertoli cell-B cell interactions may be uncovered.

## Therapeutic Applications of Sertoli Cells

Sertoli cells generate and maintain an immunoregulatory environment within the testes conducive to spermatogenesis and germ cell survival. In addition, it is well-known that their ability to regulate the immune response is not limited to the testes [**Table 2**, extensively reviewed in (18)], which makes them attractive candidates for the treatment of various health issues such as transplantation and autoimmune disease [extensively reviewed in (18)]. For instance, Sertoli cells have been shown to improve symptoms and even reverse the disease state in animal models of type 1 and 2 diabetes (108–117, 132); neurodegenerative diseases such as Parkinson’s disease (118, 119), amyotrophic lateral sclerosis (121), Alzheimer’s disease (125), and Huntington’s disease (122, 124); Laron syndrome (130); male infertility (14); and muscular dystrophy (133–135). Most of these studies included transplantation of Sertoli cells either alone or with co-grafted cells, tissues, or organs. For co-transplantation, Sertoli cells have been shown to prolong survival of allogeneic pancreatic islets, renal cells and skin grafts, xenogeneic islets, neurons, adrenal chromaffin cells, liver cells and skin grafts, and even provide prolonged survival of allogeneic heart and kidney organs. Additionally, Sertoli cells have been found to protect syngeneic islets from autoimmune destruction after transplantation into non-obese diabetic mice (111, 136) and another study found encapsulated neonatal porcine Sertoli cells could prevent and reverse the onset of autoimmune diabetes in non-obese diabetic mice (101).

**Table 2:** SC therapeutics.

Illness	Treatment	Results	Ref
**TIDM**	Rat SC co-transplanted with islets into diabetic rats	Transplant survival and normoglycemia 100+ days post-transplantation	(108, 109)
**TIDM**	Rat SC co-encapsulated with fish islets transplanted into diabetic mice	Prolonged graft survival and normoglycemia > 64 days	(110)
**TIDM**	Syngeneic mouse SC transplanted under right kidney capsule and syngeneic islets under left kidney capsule of non-obese diabetic mice	SC elicited systemic tolerance, 64% of recipients normoglycemic for 60+ days	(111)
**TIDM**	Pig SC encapsulated and injected by IP into non-obese diabetic mice	Reverted of TIDM, islet regeneration	(101)
**TIDM**	Pig SC and pig islets infused into collagen mesh transplanted in upper anterior wall subcutaneously of adolescent TIDM patients	50% recipients had at least a 50% reduction in exogenous insulin requirement for 3+ years	(112)
**TIDM**	Mouse or pig SC engineered with adenoviral or lentiviral vector to produce human insulin were transplanted into diabetic mice or rats, respectively	Normoglycemia restoration for 1-4 and 46-50 days post-transplantation, transplanted SC produced insulin and survived throughout study	(113–115)
**TIDM**	EC cultured in high glucose + SC conditioned media from SC engineered to produce insulin and C-peptide	Reduction in vasoactive substances and reactive oxygen species	(116)
**TIIDM**	Encapsulated pig SC were transplanted into subcutaneous fat of db/db diabetic mice	Decreased adipose inflammation, normalization of glucose in 60% of recipients	(117)
**Parkinson’s Disease**	SC co-transplanted with dopaminergic fetal rat or mouse neurons into 6-hydroxy-dopamine treated rats (Parkinson’s model)	SC co-transplants had an increase of 20% viability and survival over controls	(118, 119)
**Cerebellar ataxia**	SC transplanted into both cerebellar hemispheres of 3-AP induced cerebellar ataxia rats	Improved motor coordination, reduced necroptosis, decreased microglial proliferation	(120)
**Amyotrophic Lateral Sclerosis**	SC transplanted into spinal cord of ALS-model mice	Reduced motor neuron degradation, improved neuronal survival, slowed disease progression	(121)
**Huntington Disease**	PC12 cells were exposed to hydrogen peroxide and SC-CM	Protected against hydrogen peroxide, increased cell viability, continued neurite growth	(122, 123)
**Huntington Disease**	Rat SC transplanted into striatum of 3-nitropropionic acid induced Huntington’s rats or mice	Decreased inflammatory cytokines, increased dendritic length, improved striatum volume, delayed disease progression	(122, 124)
**Alzheimer’s Disease**	Bilateral hippocampal transplant of SC into rats with β-amyloid legions	Decreased neuronal cell death, improved learning and memory	(125)
**Spinal cord injury**	SC engineered to produce human neurotrophin-3 were used to condition media, cortical neurons cultured in SC-CM	Increased neurite growth	(126)
**Spinal cord injury**	SC engineered to produce human neurotrophin-3 were transplanted into rat injured spinal cord	Reduced macrophage infiltration, decreased inflammation, SC survived 42+ days post-transplantation	(126)
**Spermato-genesis defects**	Healthy mouse SC were injected into testes of *Sl/Sl^d^* SC-defect mice	92% recipient testis had SC-tubules; 62% recipient testis had normal spermatogenesis	(127)
**Spermato-genesis defects**	SC from *W/W^v^* GC-defect mice were transplanted into testes of *Sl/Sl^d^* SC-defect mice	Rescued spermatogonia, spermatocytes, round spermatids, and some elongated spermatids, some restoration of spermatogenesis	(128)
**Spermato-genesis defects**	Normal SCs transplanted into rats with irradiated testes	SC colonized seminiferous tubules, created tubule structures, recovered endogenous spermatogenesis	(129)
**Male infertility**	Transplantation of allogeneic spermatogonial stem cells into testes of NANOS2 knockout animals	Allogeneic germ cell engraftment; donor-derived spermatogenesis in mice, goats, and pigs	(14)
**Laron Syndrome**	Pig SC were encapsulated and transplanted into GHR-/- mice	Proportional growth, increased growth	(130)
**Muscular Dystrophy**	SC were encapsulated and IP injected into *mdx* mice	Increased muscle performance, improved muscle tissue morphology, decreased inflammation	
**Deep lung respiratory issues**	SC containing curcumin (anti-inflammatory) nanoparticles were injected into lateral tail vein of mice with pulmonary perivascular inflammation	90% of curcumin delivered to deep lung, improved lung inflammation	(131)

ALS, amyotrophic lateral sclerosis; EC, endothelial cell; GC, germ cells; GHR-/-, growth hormone receptor-knockout; IP, intraperitoneal; mdx, nonsense point mutation to prevent functional dystrophin production; PC12, neuron-like rat pheochromocytoma cell line; TIDM, type I diabetes mellitus; SC, Sertoli cell; SC-CM, Sertoli cell conditioned media; Sl/Sl^d^, Steel/Steel^dickie^; TIIDM, type II diabetes mellitus; W/W^v^, white spotting mice.

Transplantation of human pancreatic islets has the potential to cure type 1 diabetes. In humans, the current acceptable clinical site for islet transplantation is hepatic infusion *via* the portal vein. A majority of the Sertoli cell-islet co-transplantation studies utilized the renal subcapsular site to co-localize these cells and thus whether Sertoli cells can protect islets infused into the liver *via* the portal vein is not well documented. Takemoto et. al., utilized a hanging drop method to prepare aggregates of Sertoli cells and islets for transplantation into liver through the portal vein (137). In this study, Sertoli cells and islets were mixed randomly initially but after 12 hours of co-culture the aggregates consisted of a Sertoli cell core with islets surrounding the core. Allogeneic co-aggregates of Sertoli cells and islets (800 co-aggregates consisting of 1.2 million Sertoli cells and islet cells from 400 islets) were transplanted into the livers of diabetic C57BL/6 mice through the portal vein without any immunosuppressive therapy. Both control (animals transplanted with islets alone) and Sertoli cell-islet co-aggregate transplanted mice, exhibited normoglycemia at day 1 post-transplantation. Furthermore, in 85.7% (6 out of 7) of the Sertoli cell-islet transplanted animals the blood glucose levels remained lower than the preoperative levels for over 100 days whereas in control animals the blood glucose returned to preoperative level around day 10 post-transplantation indicating islet cell loss. Histological analysis of the transplanted cells further confirmed this as very few insulin-positive cells were detected in control animals at day 14 whereas abundant insulin-positive cells were detected in the liver of Sertoli cell-islet co-transplanted mice at 120 days post-transplantation despite the infiltration of lymphocytes into the grafts. Collectively, these data indicate that Sertoli cells have the potential to protect islets at a clinically accepted human transplant site (137).

While the exact mechanisms by which Sertoli cells protect the co-grafted cells are not completely known, it is generally believed that this protection is due to the production of immunomodulatory factors by Sertoli cells that decrease inflammation and promote immunoregulatory cells. For instance, a study by Jhao YT et al. (119), demonstrated that co-transplantation of Sertoli cells with fetal allogeneic or xenogeneic (porcine) ventral mesencephalic (VM) tissue into the striatum of a Parkinson’s disease rat model significantly improved drug-induced rotational behavior, enhanced maturation of the transplanted dopaminergic cells and their survival as compared to controls transplanted with VM alone. Analysis of the immune response in grafted striatum revealed that co-transplantation of Sertoli cells with allogeneic or xenogeneic VM tissue resulted in lower ratio of OX6+ (activated microglial cell marker)/Iba1+ (total microglia marker) cells as compared to controls indicating that Sertoli cells attenuated activated microglia in these animals. Additionally, CD3 T cell infiltration was significantly decreased in Sertoli cell co-grated animals as compared to controls (119).

The plethora of factors secreted by Sertoli cells along with their ability to survive as allografts or xenografts without the use of immune suppression, has led to their possible use as drug delivery vehicles. In addition to their ability to provide endogenous factors, such as insulin-like growth factor-1 for treatment of Laron syndrome (130), Sertoli cells have been genetically engineered to deliver insulin for treatment of diabetes and neurotrophin-3 to treat spinal cord injury (113, 126). Sertoli cells have also been modified using nanoparticles to deliver the anti-inflammatory compound curcumin to the deep lungs to treat lung inflammation (131).

Related to male fertility, spermatogenesis has been restored in Steel/Steel^dickie^ (Sl/Sl^d^) mice by engineering Sertoli cells with a lentivirus containing the Sl gene (138). These Sl/Sl^d^ mice are infertile due to a mutation in the *Sl* gene in Sertoli cells that leads to the absence of stem cell factor (also known as c-kit ligand) and a lack of spermatogenesis. Stem cell factor binds germ cell c-kit tyrosine kinase receptor, which is necessary for spermatogonial cell proliferation. Injection of the lentivirus containing the *Sl* gene into the testis resulted in mice with morphologically normal tubules containing spermatozoa and motile sperm in the epididymis. Implantation of pseudo-pregnant mice with oocytes microinseminated with late-stage spermatids or spermatozoa collected from the engineered testis led to normal offspring. More recently, CRISPER-Cas9 technology was utilized to generate NONOS2 knockout mice, pigs, goats, and cattle (14). Loss of NANOS2 leads to male, but not female, infertility due to germline ablation. Allogeneic transplantation of male spermatogonial stem cells isolated from mouse, pig, and goats into testes in NANOS2 knockout animals led to allogeneic germ cell engraftment and donor derived spermatogenesis (14). In mice and cattle, it resulted in production of donor derive offspring (unpublished data by Jon Oatley, Washington State University) (14). Use of this technology has potential for breeding desired traits in livestock or preservation of endangered species. In another study using cryopreserved testicular tissue from Rhesus macaques, autologous transplantation subcutaneously led to mature sperm production capable of generating a baby nonhuman primate (139). Thus, autologous transplantation of cryopreserved testis tissue has potential as a treatment to prevent infertility for prepubertal cancer patients who will undergo chemotherapy that will kill their germ cells. Survival and development of the transplanted tissue is especially interesting given the auto-immunogenic nature of the transplanted germ cells. Collectively, these studies support that Sertoli cells are critical for inducing an immunoprotective anti-inflammatory environment for tissue preservation and graft survival thereby supporting their role as key players in testis immune privilege.

## Sertoli Cell Response to Infections and Foreign Tissue

One downside to the immune protective environment of the testis, is that this ability to protect the germ cells from the immune system is the same mechanism that could make the testes a potential sanctuary site for infections (5, 7). Here we will discuss viral and bacterial infections in the testis as well as mechanisms used by Sertoli cells to mount an effective inflammatory antimicrobial response to prevent and combat infection.

### The Testis as a Sanctuary Site

Viruses and bacteria have been detected in the testis. At least 27 different viruses have been detected in human semen and 12 of these viruses have been detected in human testes. A review extensively describing the presence of various bacteria in the male genital tract and viruses in human semen and/or testis has been published (6), thus in this review we will focus on more recent findings. Although, bacterial infections are rare and generally cleared after antibiotic treatment, Gram*-*positive *(Enterococcus faecalis, Streptococcus pneumonae, Actinomyces neuii)* and Gram-negative (*Pseudomonas spp, Salmonella enteritdis, Neisseria gonorrhea*, *Chlamydia trachomatis, and Escherichia coli)* bacteria along with *Ureaplasma urealyticum* have been found in the male genital tract. *C. trachomatis* is the most common bacterial cause of sexually transmitted infection in humans and the only reported species of bacteria that can productively infect Sertoli cells (140, 141). In a recent study, chlamydial major outer membrane protein (MOMP) was detected in 45.3% (43 out of 95 samples) of fixed testicular biopsies taken from infertile men. A selected number of fixed testicular biopsies that were positive for MOMP also stained positive for the active replication marker TC0500. There was a 100% concordance between MOMP and TC0500 staining indicating that *C. trachomatis* was actively replicating within the testes at the time of biopsy. *C. trachomatis* DNA was detected in 16.7% (3 out of 18 samples) of fresh testicular biopsies. Interestingly, none of these patients were previously diagnosed with a sexually transmitted disease including *C. trachomatis* (142). To further understand the route by which Chlamydia travels from the initial site of infection to the testes, a mouse model of male chlamydial infection was utilized. In this model, macrophages transported the Chlamydia from the penile urethra (site of initial infection) to the testes within 3 days of infection, bypassing the entire male reproductive tract. *In vitro* data demonstrated that Sertoli cells, Leydig cells, spermatogenic cells along with testicular macrophages are susceptible to infection thereby indicating that infected macrophages from the penile urethra transfer the infection to testicular somatic and germ cells causing sperm DNA damage and impaired spermatogenesis (143).

Viral infections are more common that bacterial infections with many different viruses having been detected within the testis including adenoviruses, Epstein Barr virus, Herpes simplex viruses (HSV), mumps, human papillomavirus (HPV), Parvovirus B19, human immunodeficiency virus (HIV), human endogenous retrovirus (HERV), Ebola, Zika virus, and even severe acute respiratory syndrome-coronavirus-2 (SARS-CoV-2, COVID-19) (reviewed in (5)). If left unchecked, these infections can lead to fertility issues.

Recent viral outbreaks of Ebola and SARS-CoV-2 have increased the interest in the testis as a viral sanctuary site. Ebola and Marburg are filoviruses in the family *Filoviridae* that cause severe hemorrhagic fever with high mortality rate. Sexual transmission of these viruses suggests that the male reproductive tract, including the testis, may act as a reservoir for these viruses, thereby causing a huge interest among the scientific community to investigate how viruses establish testicular persistence and are shed in semen. Coffin KM et al., screened a collection of archived tissue samples from 97 crab-eating macaques (73 males and 24 females) that had survived experimental Marburg infection through day 47 post exposure (144). Overall, 23.7% had Marburg genomic RNA in the testes and/or eye tissue with 30.1% (22 out of 73 survivors) of the males being positive for viral genomic RNA in the testes. Marburg persistence disrupts the normal architecture of the seminiferous tubules, causing focal orchitis, germ cell loss, infiltration of inflammatory immune cells and IgG antibody deposition in the tubules suggesting persistent Marburg infection may result in partial loss of testicular immune privilege. In this study, Marburg mainly infected Sertoli cells causing detachment of these cells from the basement membrane and disruption of the BTB as indicated by the decreased expression of TJ proteins (zonula occludens (ZO)1 and TJP2). Interestingly, abundant number of CD4+Foxp3+ Tregs were detected in the testes with Marburg persistence suggesting the presence of these regulatory cells may have sustained virus infection. However, early administration of antiviral therapy prevented testicular Marburg persistence. Another study screened archived tissue samples from rhesus (n=112) or crab-eating (n=48) monkeys that were experimentally exposed to Ebola virus (145). Only 9.82% (11 out of 112) of rhesus monkeys had detectable viral genomic RNA in the eye (11.54%, 9 out of 78 tested samples), epididymis (1.32%, 1 out of 76 samples) or brain (1.25%, 1 out of 80 samples) tissues. Macrophages/monocytes served as primary reservoirs for the virus. Persistence of virus in the epididymis caused infiltration of immune cells including macrophages, eosinophils and lymphocytes. Interestingly, Ebola virus genomic RNA was not detected in any of the tissues of crab-eating monkeys. Persistence of Ebola virus was detected in 91.67% of the reproductive tissue samples, both testes and epididymides, of rhesus monkeys that died of virus infection (n=24). In 2 out of 8 animals that survived longer (16-24 days) than the typical virus exposure death frame (5-11 days), Ebola virus glycoprotein was detected in germ cells with a staining pattern suggesting Sertoli cells were also positive. Although, double immunofluorescence with a Sertoli cell marker should be performed for confirmation of Sertoli cell infection.

According to the World Health Organization, 483,556,955 confirmed cases of SARS-CoV-2 including 6,132,461 deaths have been reported to-date globally. The studies reporting presence of SARS-CoV-2 in semen showed mixed findings. Li et al., reported the presence of virus in the semen of 6 out of 38 patients using RT-PCR (146). Out of these 6 patients, 2 were in the recovery stage while 4 were in the acute stage of infection. Other studies, where a total of 262 semen samples were analyzed, failed to detect the presence of SARS-CoV2 in the semen but the sperm quality in patients with moderate infection was impaired (147). Analysis of the testis revealed the presence of viral particles in 10 out of 33 testicular samples. Detection of virus in the testis was associated with germ cell loss, deposition of IgG antibodies in the seminiferous tubules and increased immune cell infiltration (CD3 T cells and CD68 macrophages) in the testicular interstitium suggesting that SARS-CoV-2 may cause impaired spermatogenesis thereby leading to male infertility (147). Given the variability among studies, a more thorough analysis of whether testis may provide a sanctuary site to SARS-CoV-2 needs to be carried out.

### BTB in Infection and Inflammation

The BTB TJ are composed of occludin, claudins, ZO, (also known as TJ protein 1, TJP1) and junctional adhesion molecules. Of these, occludin and claudin 11 are the most critical for BTB integrity and male mice lacking these proteins are infertile (148–151). Disruption of occludin or claudin 11 results in loss of BTB integrity, increased permeability of the BTB and germ cell loss. Interestingly, in these mice, autoantibodies were not detected (148, 149), although this could be due to the loss of germ cells before an immune response occurs. Moreover, normal humans and guinea pigs lack occludin in their Sertoli cell TJ and are fertile (152).

Cytokines such as TNF-α and TGF-β2/3 reversibly disrupt the BTB [reviewed in (153)]. Under normal conditions this plays an important part in how germ cells cross from the basal to the adluminal compartment of the seminiferous epithelium without compromising BTB integrity. As preleptotene spermatocytes transit across the BTB, these cytokines promote the disassembly of the old BTB above the spermatocytes. At the same time testosterone promotes the formation of a new BTB below the spermatocytes affectively creating a transient intermediate compartment that allows the germ cells to cross the BTB without exposing the adluminal compartment.

However, under pathological conditions inflammation is associated with male infertility and abnormal expression of cytokines could affect TJ proteins and the BTB. For example, experimental autoimmune orchitis (EAO) is associated with increased IL-6 and TNF-α. Using an EAO rat model, it was found that EAO increased leukocyte infiltration, altered the localization of TJ proteins (occludin, claudin 11 and TJP1) and increased BTB permeability (154). Similar results were observed in rats treated with IL-6 and *in vitro* IL-6 and TNF-α disrupted Sertoli cell BTB function.

Consistently, testicular viral infection initiates an inflammatory response that disrupts BTB permeability. Mumps virus infects Sertoli cells leading to toll-like receptor (TLR)2 activation and increased TNF-α production. This decreases occludin and ZO1 expression leading to disruption of BTB integrity (155). Similarly, Marburg virus infection of the testis leads to immune cell infiltration, including lymphocytes (T cells and B cells), macrophages and neutrophils, and IgG deposition in the interstitium and seminiferous tubules (144). Infection starts in the interstitial cells and then progresses to the seminiferous tubules including peritubular myoid cells, Sertoli cells and germ cells. Sertoli cell infection results in a decrease in TJ protein expression, BTB disruption and germ cell loss. In surviving macaques, Sertoli cells were the primary reservoir and Tregs were associated with infected tubules. SARS-CoV-2 infection affects the testis, with an increase in inflammatory cytokines (TNF-α, IL-6 and IL-1β), a decrease in TJ proteins (occludin and claudin 11) and an altered spatial organization of testicular cells (156). Zika virus has been shown to infect and replicate in human Sertoli cells *in vitro* and while infection did not affect barrier permeability, virus was released from both the basal and adluminal surface of Sertoli cells, indicating the potential for the virus to enter the seminiferous tubules and be sexually transmitted through the male reproductive tract (157). Indeed, Zika virus is sexually transmitted (5). Another route for Zika viral transmission is infection of spermatogenic germ cells and macrophages (158). Here circulating macrophages infected with Zika entered the testis, altered claudin 1 localization and increased BTB permeability, allowing for macrophage invasion of the seminiferous tubules. At the same time the germ cells were destroyed. Zika virus also infects human macrophages and culture of human Sertoli cells with supernatant collected from these macrophages increased Sertoli cell barrier permeability (157). HIV-1 shedding in semen of antiretroviral treated men is detected despite undetectable levels of virus RNA in the blood suggesting persistence of the virus in the male genital tract. Using an *in vitro* rat Sertoli cell model that mimics the BTB, it was demonstrated that HIV-1 infection disrupts the BTB by perturbing the distribution of TJ (e.g., occludin, ZO1) and basal ectoplasmic specialization (e.g., N-cadherin, and β-catenin) proteins. Additionally, the organization of actin and microtubule cytoskeleton was disturbed in the Sertoli cells (159).

## Sertoli Cell Protection From Pathogens

Remarkably, Sertoli cells can also protect against pathogens by initiating an antimicrobial response. Sertoli cells express a wide range of pattern recognition receptors (PRRs) that can identify and react to a wide range of pathogens (160, 161). When PRRs bind to a pathogenic marker, they initiate signaling cascades, like NF-κB, that allow Sertoli cells to express and secrete various antimicrobial molecules such as IL-1β, IL-6, TNF-α, MCP-1, activin A, protein kinase R (PKR), and β-defensin (**Table 1**) (23, 58, 73). This can recruit and activate immune cells such as M1 macrophages and T cells.

### Pattern Recognition Receptors

TLRs are a type of PRR, usually extracellular, that recognize microbial and viral markers to allow for further immune response. Specifically, TLRs recognize pattern-associated molecular patterns (PAMPs) that are not expressed by host cells such as: cell wall subunits, LPS components of membranes, flagellin proteins, and viral proteins (162). Upon PAMP recognition, TLRs become activated and trigger signaling events that elicit inflammatory responses and immune cell maturation in order to eliminate the pathogenic threat (162). So far, 10 different TLRs have been identified in humans, and each have different discriminations for the various PAMPs they recognize (**Table 3**). TLR1, TLR2, TLR4, and TLR6 recognize bacterial proteins to trigger responses against invading bacteria pathogens (163, 164, 166, 168). TLR3 mainly recognizes viral dsRNA, while TLR10 recognizes viral and bacterial dsRNA (165, 172). TLR5 recognizes components of bacterial flagella (167). TLR7 and TLR8 recognize ssRNA viral particles (169, 170). TLR9 recognizes unmethylated CpG DNA (171).

**Table 3:** Various PRRs and their ligands.

PRR	Ligand	Ref
**TLR1**	Gram-positive bacterial components (triacyl lipopeptides, diacyl lipopeptides, GPI anchors, peptidoglycans, and lipoproteins)	(163)
TLR2*	Gram-positive bacterial components (peptidoglycans, lipopeptides, and lipoproteins)	(164)
TLR3*	Viral dsRNA	(165)
TLR4*	Gram-positive and gram-negative bacterial components (LPS, peptidoglycans, lipopeptides, and lipoproteins)	(166)
TLR5*	Flagellin of bacteria	(167)
TLR6*	Gram-positive bacterial components (triacyl lipopeptides, diacyl lipopeptides, GPI anchors, peptidoglycans, and lipoproteins)	(168)
**TLR7**	Viral ssRNA and dsRNA	(169)
**TLR8**	Viral and bacterial ssRNA and dsRNA degradation products	(170)
**TLR9**	Unmethylated CpG DNA components	(171)
**TLR10**	Viral and bacterial dsRNA and other products	(172)
NOD1*	Mainly gram-negative bacteria amino acids	(173)
**NOD2**	Muramyl dipeptides of bacterial peptidoglycans	(173)

* Indicates expression by SC (160).

Of these TLRs, Sertoli cells express TLR2, TLR3, TLR4, TLR5, and TLR6 (160, 161). Expression of TLRs such as TLR2, TLR4, TLR5, and TLR 6 would elicit a powerful defensive response against bacterial invasion, with TLR3 expression allowing for an antiviral response. TLRs activate the NF-κB signaling pathway in Sertoli cells, which upregulates their production of ICAM-1, MCP-1, IL-6, IL-1β, and TNF-α (160). As a cellular adhesion molecule, intracellular adhesion molecule 1 (ICAM-1) allows lymphocytes to bind and migrate through the area (160). Sertoli cell ICAM-1 has been shown to be an important regulator of spermatogenesis by regulating the interactions between the BTB and the apical ectoplasmic specialization (174). MCP-1 encourages chemotaxis and migration of monocytes (160). In the testes, IL-1 has been demonstrated in the maintenance of spermatogenesis (58). IL-1β also operates through NF-κB signaling in macrophages, neutrophils, and T cells to cause expression of other proinflammatory molecules such as TNF-α and IL-6 (23, 58, 73). On T cells in particular, IL-1β induces the generation of proinflammatory Th17 cells (**Figure 4**) (73). IL-1β can lead to expression of TNF-α. TNF-α is an inflammatory cytokine that leads to apoptosis or necrosis of cells (74). Conversely, in the testis TNF-α can promote cell survival (75). In conjunction with TGF-β, TNF-α also regulates the BTB in spermatogenesis, allowing preleptotene and leptotene spermatocytes to migrate across the barrier (76). The cytokine IL-6 promotes a proinflammatory response, thus allowing for adequate destruction of the pathogen (**Figure 4**) (160). Moreover, IL-6 expression by Sertoli cells has been shown to mediate signaling cascades that are essential for spermatogenesis (58, 71). IL-6 plays a dual role in immunity depending on the environment. Normally, IL-6 stimulates responses to infection and tissue damage by increasing expression of acute phase proteins, production of platelets, B cell production of antibodies, and leads T cells to differentiate into effector subtypes Th17 and CTLs (72). However, in the presence of TGF-β, retinoic acid (RA), and dendritic cells, IL-6 can generate Tregs (175). Interestingly, Sertoli cells also express TGF- β and store retinoic acid (**Figure 4**).

Altogether, Sertoli cell TLR engagement leads to an antibacterial and antiviral response through inflammatory mechanisms. If these reactions are appropriately regulated, the response would be enough to eliminate the pathogen while maintaining a germ cell-healthy environment. If not sufficiently regulated, this inflammatory response could disrupt the seminiferous tubules and lead to germ cell destruction.

One-way Sertoli cells can regulate TLR activation is through Tyro3, Axl, and Mer (TAM) receptors. TAMs play an important role in the trophic support of germ cells when activated by the ligands growth arrest-specific gene 6 (Gas6) and protein S (176, 177). Triple TAM knockout male mice displayed small testes with no sperm once in adulthood, among many other side effects (176). Initially, the testes developed normally though the juvenile stage, but quickly afterwards significantly fewer spermatocytes were detected (176). Although Sertoli cells appeared normal, gametes were not detectable in adults implying that apoptosis of the germ cells was not due to a Sertoli cell issue, but instead could be attributed to a lack of trophic support established by TAM (176). In addition to their role in spermatogenesis, TAMs act as a negative regulator of the TLR3 and TLR4 signaling pathway (178). Gas6 interaction with TAM leads to increased expression of TLR signaling suppressors such as suppressor of cytokine signaling (SOCS) 1 and SOCS3 (178, 179). In Sertoli cells, SOCS1 and SOCS3 activate the STAT1 signaling pathway to decrease inflammatory responses and increase clearance of apoptotic cells through phagocytosis by cells such as macrophages and dendritic cells (178).

In addition to TLRs serving mainly as extracellular PRRs, NOD-like receptors (NLRs) serve as intracellular PRRs. NLRs sense various PAMPs within the cell, and once activated, initiate signaling cascades leading to proinflammatory responses. Sertoli cells express different NLRs including NOD1 and NOD2 (**Table 3**) (173). NOD1 senses specific amino acids mainly related to gram-negative and some gram-positive bacteria. Upon recognition, NOD1 increases production of IL-1β and inhibits maturation of autophagosomes (173). NOD2 recognizes muramyl dipeptides peptidoglycans present on bacteria, which activates caspase-1, leading to IL-1β secretion and maturation of autophagosomes (173, 180). While activation of NLRs in SC initially leads to expression of pro-IL-1β, upon Sertoli cell development, NOD2 decreases in the ability to express IL-1β (173). As TAM regulates TLR activation, it also plays a role in restricting NLR-derived production of proinflammatory cytokines TNF, IFNs, IL-1β, and IL-6 (173). Again, this is another example of how Sertoli cells maintain a balance in the testis between an antimicrobial response to infection and a regulatory response to shield immunogenic germ cells.

### Sertoli Cell Antimicrobial Response and Delivery of Antibiotics

β-defensins are antimicrobial peptides expressed by Sertoli cells in response to bacterial infections and viral-infected cells (77, 78). IFNs are antiviral peptides that also regulate Sertoli cell survival and function (79, 81). IFNs are important in antiviral responses by activating natural killer cells and macrophages (178). IFNs can also activate and promote the function of inflammasomes within these leukocytes, aiding in clearance of viral infections (80). Regarding adaptive immunity, IFN-γ causes T cell polarization toward Th1 proinflammatory phenotype (58). IFNs are also important in increasing expression of PKR in the presence of infection by viruses (81). Sertoli cells also express the antiviral peptide PKR in response to stimulation by IFN-γ (81). The role of PKR in combating viral infections is to inhibit translation of viral mRNA and stimulate apoptosis of the infected cell (82). Furthermore, PKR also participates in a positive feedback loop with IFNs, amplifying the antiviral response (82).

These peptides can recruit immune cells like macrophages in the case of resilient and persistent infection, mounting an effective phagocytic response against pathogens in the testicular interstitium to prevent invasion of pathogens into the seminiferous tubules (181). Furthermore, Sertoli cells may be able to influence another antimicrobial and antiviral immune cell, natural killer (NK) cell in case of infection. NK cells are not commonly found in the testes but can migrate there during severe inflammation or infection (95). NK cells can mount a strong, innate response against viral infected cells and other microbials by secreting proinflammatory molecules and through cytolysis (182). Interestingly, when NK cells (human, mouse, or guinea pig) were co-cultured with 20-day old rat Sertoli cells, their inflammatory functions were inhibited in a dose-dependent way, but their ability to lyse cells was not affected (183). This implies that Sertoli cells may be able to modulate their inflammatory response to prevent collateral damage while still allowing NK cells to fight infection.

As part of the normal process of spermatogenesis, Sertoli cells phagocytose apoptotic germ cells and residual bodies. These phagocytic abilities also allow Sertoli cells to phagocytose bacteria. However, phagocytosis of *Staphylococcus aureus* by Sertoli cells differed from macrophage phagocytosis in that Sertoli cells used alpha-defensin to kill the bacteria without eliciting an inflammatory response. However, this was not as effective at killing the bacteria as macrophage phagocytosis (184). Taking advantage of these phagocytic properties, Sertoli cells were loaded with microparticles containing the antibiotic complex ofloxacin and palladium (185). The combination of antibiotic and Sertoli cell antibacterial factors resulted in significant killing of *Pseudomonas aeruginosa*. Overall, Sertoli cells can act as a double-edged sword capable of producing immunomodulatory factors that not only protect the germ cells from an autoimmune response but that can also fight of pathogens (**Figure 4**).

## Summary

Sertoli cells are a critical component in testicular immune regulation. As such, they interact with various parts of the immune system, both innate and adaptive, to create and maintain an immunoregulatory environment within the testes conducive to spermatogenesis and germ cell survival. These very abilities make them attractive candidates in treatments and therapies for various health issues such as transplantation and autoimmune diseases. On the other hand, these abilities may create a safe haven where infectious agents are sequestered from the immune system. Interestingly, Sertoli cells can act as a double-edged sword with both the ability to create an immune protective environment and also the ability to activate innate and adaptive immune responses to fight off infections (**Figure 2**). While these roles appear contradictory we believe they are essential for normal testicular function. Further understanding the mechanisms of Sertoli cell immune regulation are important to take advantage of their therapeutic potential while at the same time minimizing any risks.

## Author Contributions

RW and TH wrote the first draft of the manuscript. GK, JD, RW, and TH wrote sections of the manuscript. All authors contributed to the conceptualization, design, revision, and approved the submitted version of this manuscript.

## Funding

This work was supported in part by NIAID grant AI109398 (to JD) and The CH Foundation (to JD and GK).

## Conflict of Interest

JD has stock in Sernova, Corp.

The remaining authors declare that the research was conducted in the absence of any commercial or financial relationships that could be construed as a potential conflict of interest.

## Publisher’s Note

All claims expressed in this article are solely those of the authors and do not necessarily represent those of their affiliated organizations, or those of the publisher, the editors and the reviewers. Any product that may be evaluated in this article, or claim that may be made by its manufacturer, is not guaranteed or endorsed by the publisher.
